# Supplementary material for: Magnetic monopole density and antiferromagnetic domain control in spin-ice iridates
Source: Nat Commun. 2022 Jan 21;13:444. doi: 10.1038/s41467-022-27964-y (PMC8782874; doi:10.1038/s41467-022-27964-y)
Supplement: Supplementary file 1 — Supplementary Information [file 41467_2022_27964_MOESM1_ESM.pdf]

Supplementary Information accompanying: "Magnetic monopole density and antiferromagnetic domain control in spin-ice iridates"

M. J. Pearce,[1,2] K. Götze,[1,3] A. Szabó,[4,2,5] T. S. Sikkenk,[4,6] M. R. Lees,[1]
A. T. Boothroyd,[2] D. Prabhakaran,[2] C. Castelnovo,[4,*] and P. A. Goddard[1,†]

[1]*Department of Physics, University of Warwick, Coventry, CV4 7AL, UK.*
[2]*Department of Physics, University of Oxford, Clarendon Laboratory, Oxford, OX1 3PU, UK.*
[3]*Deutsches Elektronen-Synchrotron (DESY), 22607 Hamburg, Germany.*
[4]*T.C.M. Group, Cavendish Laboratory, J. J. Thomson Avenue, University of Cambridge, Cambridge, CB3 0HE, UK.*
[5]*ISIS Facility, Rutherford Appleton Laboratory, Harwell Campus, Didcot, OX11 0QX, UK.*
[6]*Institute for Theoretical Physics and Center for Extreme Matter and Emergent Phenomena, Utrecht University, Leuvenlaan 4, 3584 CE Utrecht, The Netherlands.*

(Dated: January 13, 2022)

I. APPLIED MAGNETIC FIELD PARALLEL TO [100]: FULL TEMPERATURE DEPENDENCE

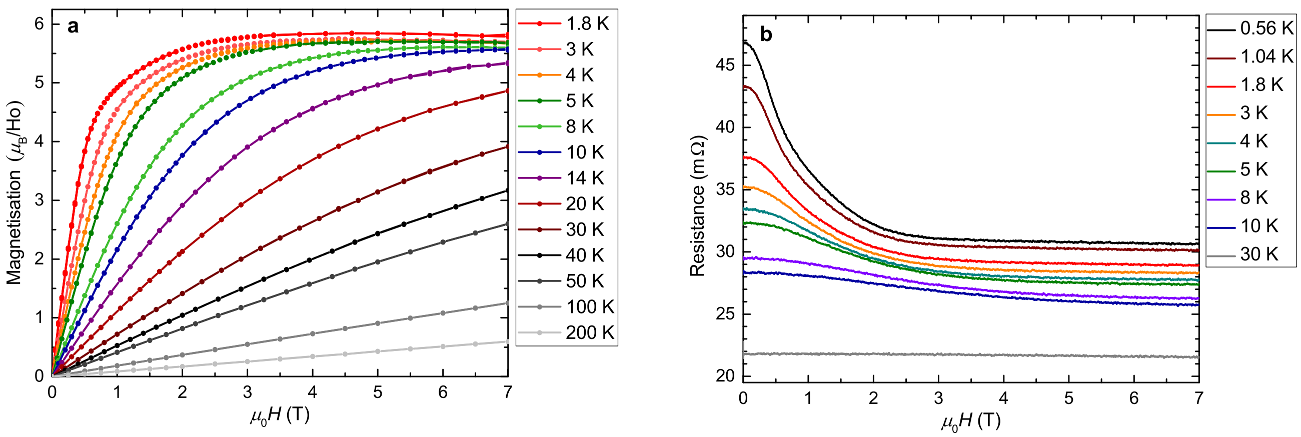

FIG. 1. Measurements of (a) the magnetisation and (b) the resistance of $Ho_2Ir_2O_7$ under an applied [100] magnetic field at various temperatures. In the main text the evolution of the magnetisation and resistance as the temperature is increased from 1.8 K to 10 K is discussed; this behaviour continues for the higher temperature data presented here.

* cc726@cam.ac.uk
† p.goddard@warwick.ac.uk

II. VARIABLE FIELD SWEEP RATE MAGNETISATION MEASUREMENTS

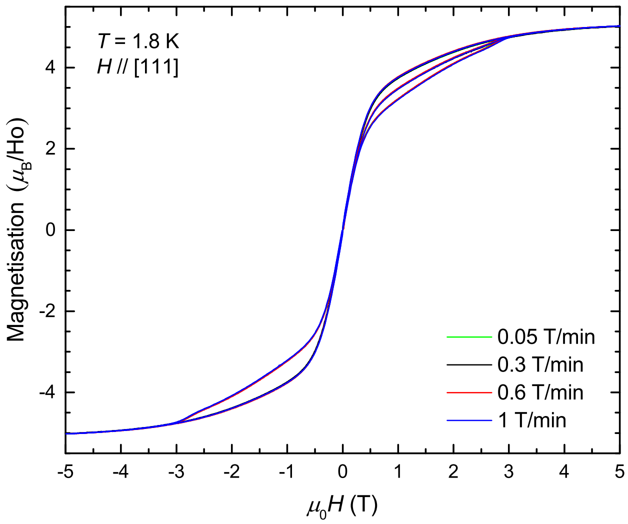

FIG. 2. Magnetisation of $Ho_2Ir_2O_7$ at 1.8 K under an applied [111] magnetic field swept at various rates. The hysteresis is insensitive to the sweep rate of the magnetic field across two orders of magnitude. Within experimental uncertainty, there is no significant broadening of the hysteresis as the sweep rate is increased, nor does it begin to open at zero applied field. This indicates that the hysteresis is static on the timescales of our experiments. A similar insensitivity to the magnetic field sweep rate was found for measurements of the magnetoresistance.

III. APPLIED MAGNETIC FIELD PARALLEL TO [111]: FULL TEMPERATURE DEPENDENCE

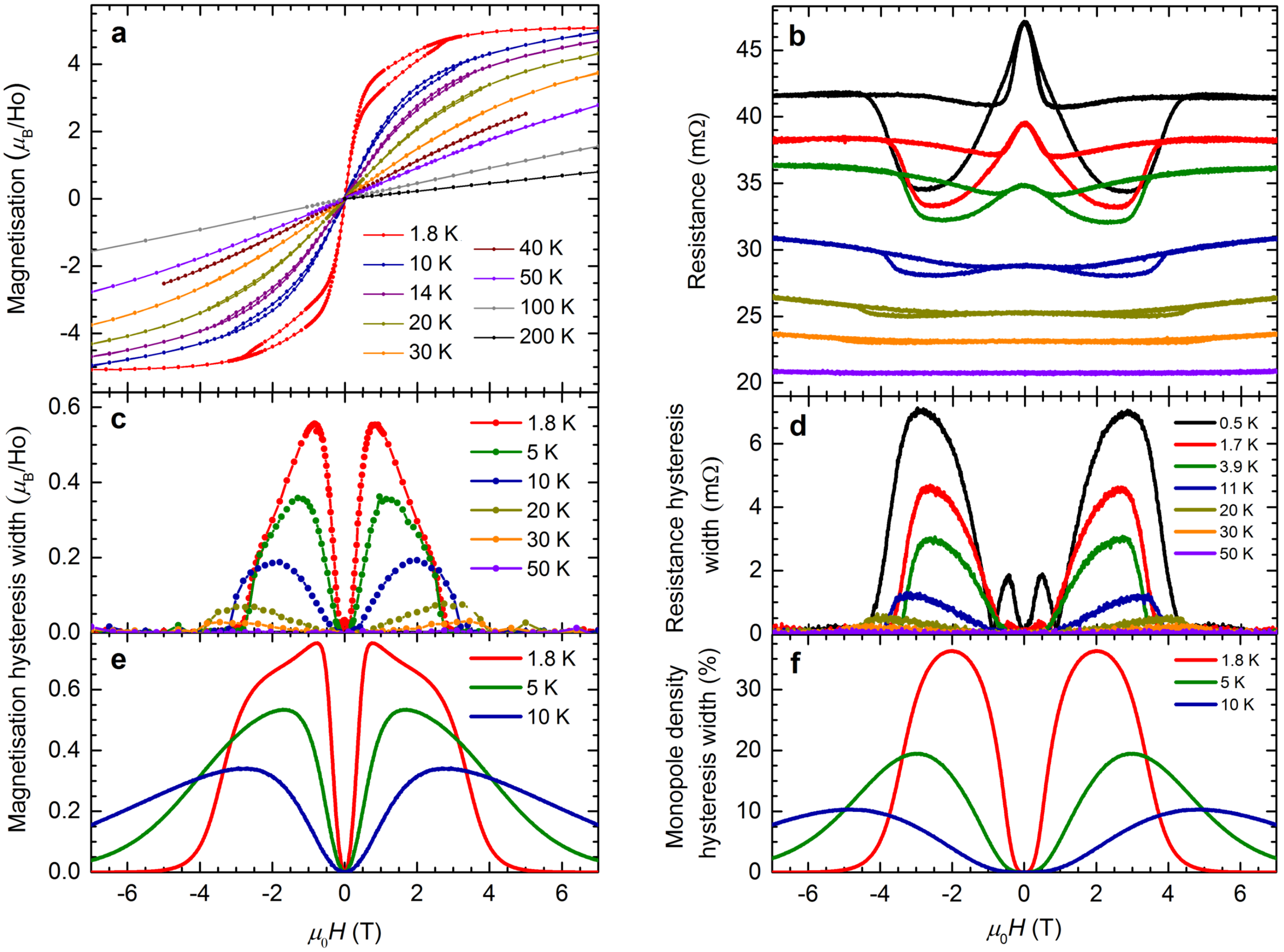

FIG. 3. Measurements of (a) the magnetisation and (b) the resistance of $Ho_2Ir_2O_7$ under an applied [111] magnetic field at various temperatures. The initial field sweep has been omitted from all traces for clarity. The width of the hysteresis, defined as the absolute value of the difference between the data measured on increasing and decreasing fields, is shown for a selection of temperatures for measurements of (c) the magnetisation and (d) the resistance and for Monte Carlo simulations of (e) the magnetisation and (f) the monopole density. The simulated hysteresis widths are calculated using a 30:70/70:30 ratio of Ir domains for -7 T to 7 T/7 T to -7 T, respectively, as this ratio gives the best agreement of the magnitude of the hysteresis width with experiments. The shape of the experimental hysteresis width is well reproduced by the simulations. We note the possibility that the resistance measurement at 0.5 K may be out of equilibrium; however we include it here for completeness.

IV. CONSIDERATIONS ABOUT DOMAIN WALL PINNING AND DRIVING

A. Driving mechanism and pinning energy scales

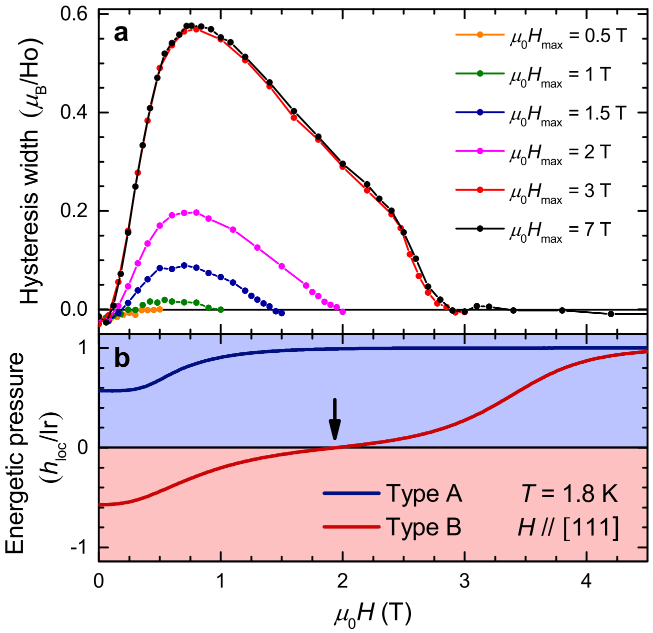

FIG. 4. **Truncated magnetisation hysteresis loops.** (a) Width of the hysteresis in the magnetisation under an applied [111] magnetic field at 1.8 K, defined here as the magnetisation measured on the downsweep minus data measured on the upsweep (excluding the virgin curve). For each magnetisation loop the magnetic field was swept from 0 to $H_{\max}$ to $-H_{\max}$ to $H_{\max}$ to 0 starting from a zero-field cooled initial condition. The hysteresis width increases with $H_{\max}$ until $\mu_0 H_{\max} \approx 3$ T, beyond which it does not open any further. We note that the narrow region of negative hysteresis width as the applied magnetic field is swept through 0 T is an experimental artefact arising due to flux pinning in the superconducting magnet used to provide the magnetic field. (b) The simulated energetic pressure applied by the Ho moments in equilibrium inside both type A and type B Ir domains for $H \parallel [111]$ at 1.8 K. The background shading indicates which domain type the energetic pressure favours: A (light blue) or B (light red). Above 2 T (arrow) the energetic pressure becomes positive inside type-B domains and it becomes favourable for them to flip; below this field both domains remain metastable.

In our work, we found evidence of plastic behaviour of the antiferromagnetic Ir domains on the time scales of our experiments. We propose that this domain wall movement is driven by their coupling to the Ho moments, specifically via the energy difference that a [111] saturated Ho configuration induces between the Ir type A and B domains. Within each domain, this energy difference is $2h_{\mathrm{loc}}\langle\sigma\rangle$ per Ir ion, where $\langle\sigma\rangle$ is the thermodynamic expectation value of the average of all Ho Ising variables σ_i in equation (1) of the main text, since the Ho–Ir interaction energy in type A and B domains is $\pm h_{\mathrm{loc}} \sum_i \sigma_i$, respectively. This energy difference is plotted in Figure 4b. For small external fields, $\langle\sigma\rangle$ is controlled by the Ho–Ir coupling and, as such, it favours the existing domain locally. While type A domains become energetically favourable in equilibrium for arbitrarily small positive (in our labelling convention) fields, they can only grow if holmium moments rearrange inside a type B domain to favour them locally: for small fields this only happens by slow thermal fluctuations of the moments over an energy barrier, which slows down domain wall movement substantially. Above a certain field (around 2 T at 1.8 K), however, $\langle\sigma\rangle$ also becomes positive inside type B domains: after this point, the growth of type A domains is energetically favoured everywhere and so it can occur via ultrafast Ir dynamics, only hindered by domain wall pinning.

Concurrently with this interpretation, our experiments show that the hysteresis starts to open significantly only when the external field exceeds 2 T (Figure 4a). It is also seen in Figure 4a that once $\mu_0 H_{\max}$ exceeds ≈ 3 T the hysteresis width does not increase any further and the observed hysteresis loops close around 2.9 T, well below the saturation of the energetic pressure that flips type-B domains. This suggests that the domain wall pinning overcome by the Ho-mediated energetic pressure is due to weak pinning sites (or potentially self-pinning of the complex magnetic structure), with net pinning energies on the order of 1 K per Ir ion. This also helps explain the absence of long tails

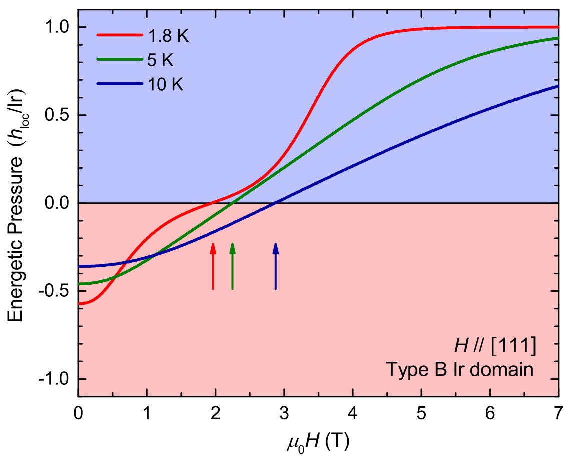

FIG. 5. **Evolution of the energetic pressure with temperature**. Energetic pressure $2h_{\text{loc}}\langle\sigma\rangle$ under an applied [111] magnetic field in type B domains for three different temperatures. The background shading indicates which domain type the energetic pressure favours: A (light blue) or B (light red). The pressure changes sign, indicating a speedup in domain wall movement, at slightly increasing external fields (arrows). This trend matches the evolution of the field at which the experimental hysteresis loops close qualitatively (see main text Figure 3 and Supplementary Figure 3).

in the experimental hysteresis curves: the critical field increases only slightly with temperature (Figure 5), causing the experimental hysteresis curve to close abruptly at slightly higher fields.

Such a weak pinning, however, does not explain the fact that the ratio of Ir domains appears to saturate around 70:30 rather than 100:0. We speculate that this is due to rare but strong pinning sites (e.g., impurities): these give rise to relaxation time scales longer than the experimental ones, which prevent the domain distribution from becoming any more polarised. Further study is needed to fully understand and model the microscopic origin of the plastic behaviour in these materials beyond this simple energetic argument, as this is an open and interesting question of direct relevance to potential applications, such as those in spintronics.

B. Other sources of antiferromagnetic domain driving

Earlier work claimed that some control over antiferromagnetic domains by an externally applied magnetic field in related systems could be achieved via the coupling of the field to the continuous canting of the spins [1], or via domain wall magnetisation induced by topological transport [2] (see also Refs. [3–5]). These mechanisms may be active in our system, leading to a direct coupling of the applied field to the iridium domain walls. However, their effect is substantially smaller than the one identified in our work (e.g., Ref. [2] claims an effective uniform magnetisation of $10^{-3}\,\mu_{\text{B}}$ per unit cell, which gives rise to an effective coupling strength on the order of millikelvins). As a result, even if these mechanisms were active in $Ho_2Ir_2O_7$, we are confident that the field–Ir coupling mediated by the Ho moments that we have identified in our work is dominant.

V. FIELD-COOLED MAGNETISATION AND RESISTANCE MEASUREMENTS

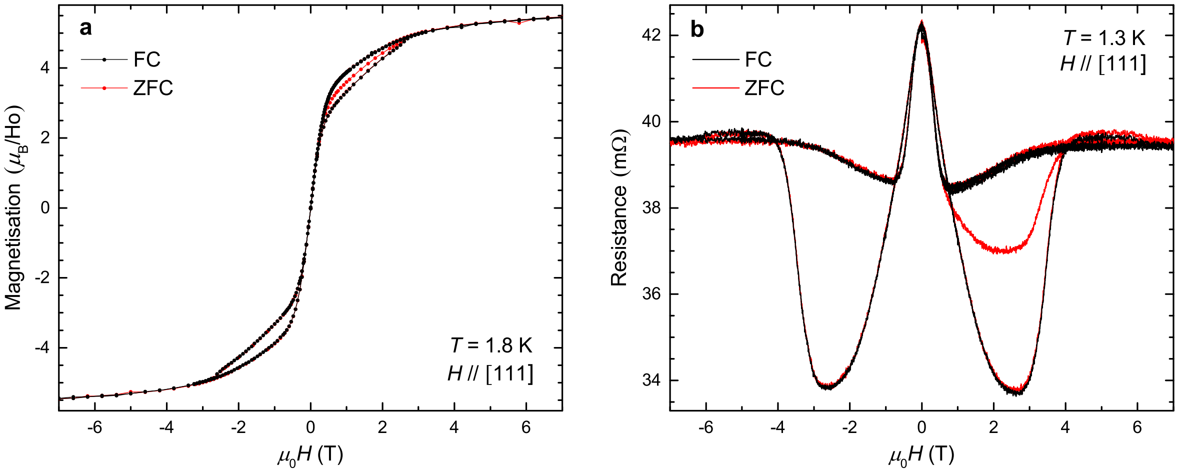

FIG. 6. (a) Magnetisation and (b) resistance under an applied [111] magnetic field measured from a field-cooled (FC) and zero-field cooled (ZFC) initial condition. ZFC magnetisation (resistance): the sample was cooled in zero applied field from 250 K (118 K) prior to measurement. FC magnetisation (resistance): the sample was cooled in a 7 T (8 T) [111] magnetic field from 250 K (54 K); once at 1.8 K (1.3 K) the field was removed and then the measurements made. The FC and ZFC measurements are identical within experimental uncertainty, except for the data recorded on the initial sweep to positive field. Upon zero-field cooling, the two Ir domains form in an approximately equal ratio, and consequently initial sweep data follow the trajectory expected for a 50:50 domain ratio (discussed in the main text, see Figure 3). By contrast, the FC initial sweep lies on top of the subsequent downsweep, consistent with the interpretation presented in the main text: cooling in a sufficiently large [111] field, plastically alters the domain ratio to approximately 70:30. It remains at this value once the field has been removed, and consequently data for the initial sweep follow the trajectory expected for a 70:30 domain ratio, rather than 50:50.

VI. APPLIED MAGNETIC FIELD PARALLEL TO [110]

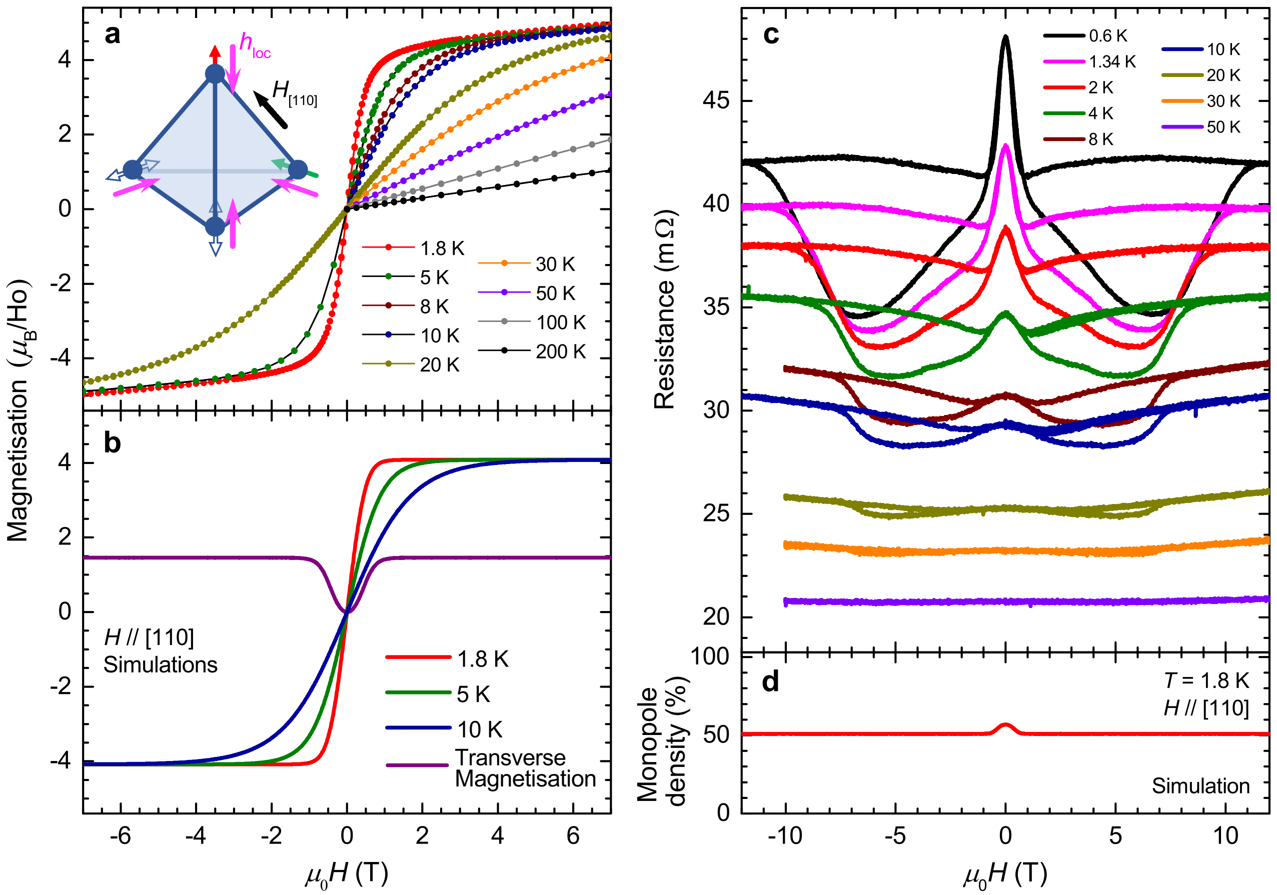

FIG. 7. **$Ho_2Ir_2O_7$ under the application of a [110] magnetic field**. (a) Measurements and (b) Monte Carlo simulations of the longitudinal magnetisation. The transverse magnetisation for a type A domain (shown here for 1.8 K in the simulations only) is the component of the magnetisation along [001] whilst the field is applied along [110]. The inset to (a) shows a single tetrahedron of the Ho^{3+} sublattice. Magenta arrows indicate the local effective field $\mathbf{h}_{\rm loc}$ due to the ordered Ir moments for a type-A domain. Under the application of an external [110] magnetic field (black arrow) one Ho moment orients parallel (green arrow) and one antiparallel (red arrow) to $\mathbf{h}_{\rm loc}$; the remaining two spins are normal to the [110] axis and thus are decoupled from the applied field. (c) Measurements of the resistance and (d) Monte Carlo simulations of the density of single monopoles. As with all other figures, demagnetisation effects have been accounted for as described in the methods section.

In this section, we present measurements of the magnetisation and magnetoresistance of $Ho_2Ir_2O_7$ under an applied [110] magnetic field which, whilst highly susceptible to misalignment, allow us to confirm some of the more subtle predictions and consequences of the theoretical interpretation presented in the main article.

The inset to Figure 7a shows that upon applying a [110] magnetic field to a type-A Ir domain, one Ho moment in each tetrahedron orients parallel to $\mathbf{h}_{\rm loc}$, one antiparallel, and the remaining two spins do not couple to the external field as they are oriented perpendicular to [110]. An equivalent configuration is adopted in type-B Ir domains, but with the direction of $\mathbf{h}_{\rm loc}$ reversed. Consequently there is no net energetic pressure, and hysteresis is not expected for this orientation.

We note that the two Ho moments which are decoupled from the external magnetic field can preferentially orient parallel to $\mathbf{h}_{\rm loc}$. While this has no effect on the longitudinal magnetisation, since the two spins are normal to [110], it

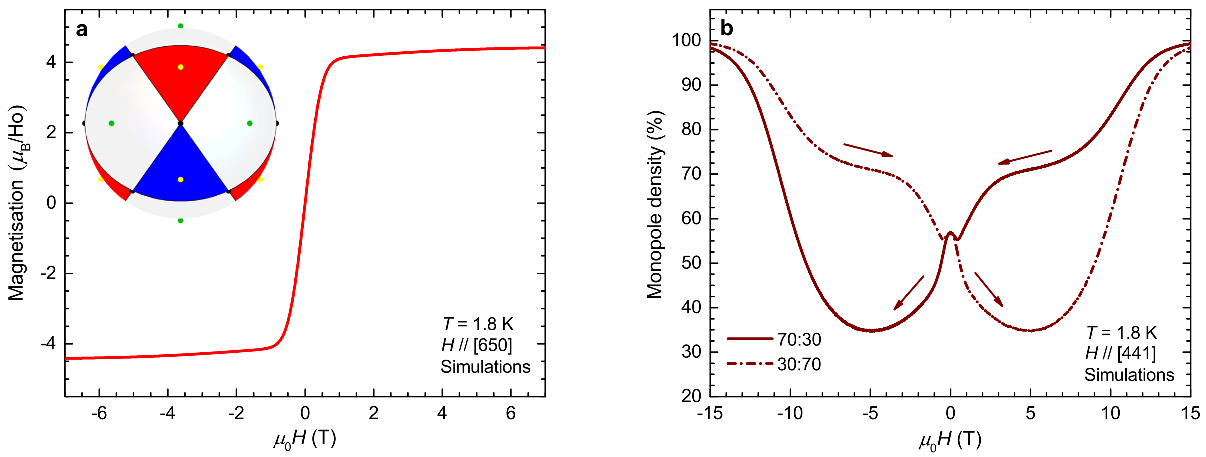

FIG. 8. **Tilted magnetisation and monopole density simulations.** Monte Carlo simulations at 1.8 K of (a) the magnetisation under an applied magnetic field along the [650] crystallographic direction, which corresponds to a tilt from [110] towards [100] of 5.19°and (b) the density of single monopoles under an applied field along [441], corresponding to a tilt from [110] towards [111] of 10.02°. The monopole density is calculated using an A:B ratio of Ir-domain types of 70:30 for 15 T to -15 T and 30:70 for -15 T to 15 T. Arrows indicate the evolution of the hysteresis loop upon sweeping the field. The tilts for the simulations were chosen as they provided a good qualitative agreement with the experimental data in Figures 7a and 7c. Inset to (a): the high-field polarised state of the Ho moments for different directions of external magnetic field, represented as a 2D projection of the applied field direction. Green, yellow, and black dots correspond to the ⟨100⟩, ⟨111⟩, and ⟨110⟩ directions, respectively. The colour coding of the regions identifies the corresponding lowest Zeeman energy state: light grey regions are 2I2O configurations and red and blue regions are 3I1O/1I3O monopole crystals.

does result in a net magnetisation along [001]. A Monte Carlo simulation of this transverse magnetisation ($M \parallel [001]$ for $H \parallel [110]$ at $T = 1.8$ K) for a type-A single-domain crystal is shown in Figure 7b (the transverse magnetisation for a type-B domain is the same curve multiplied by a factor of -1). For a multidomain crystal the bulk transverse magnetisation is expected to be non-zero except in the case of a 50:50 domain ratio (for which the contributions from the two domain types cancel), and saturates at a magnetisation which depends on $\mathbf{h}_{\mathrm{loc}}$, the temperature, and the Ir domain ratio imbalance. Notably, this transverse magnetisation (due to the Ho moments) only occurs because of the magnetism of the ordered Ir moments (via $\mathbf{h}_{\mathrm{loc}}$), and so is not expected to be present in the analogue titanate compound $Ho_2Ti_2O_7$.

The expected absence of hysteresis for $H \parallel [110]$ is apparent in Monte Carlo simulations of the magnetisation (Figure 7b) and the monopole density (Figure 7d). The simulated magnetisation saturates at 4.08 μ_B/Ho, as is expected for the spin orientation described above and the monopole density is broadly insensitive to the applied magnetic field. However, experimental measurements of the resistance (Figure 7c) show highly hysteretic behaviour which is similar in form to data measured under an applied [111] field (see main article), but closes at higher fields. The magnetisation (Figure 7a), whilst non-hysteretic, exceeds the saturation magnetisation value expected for this orientation and indeed does not saturate over the measured field range, instead retaining a positive gradient at high fields.

The divergence of the experimental results from the expected behaviour for this field direction arises due to the particular sensitivity to misalignment of the measured properties close to the [110] orientation. The inset to Figure 8a shows that the [110] order (where one spin points into the tetrahedron, one points out, and two are decoupled from the external field) is stabilised only when the applied field is perfectly aligned in that direction. Any deviation, no matter how small, couples the applied field to the two Ho spins per tetrahedron which are normal to the [110] direction. These spins can then polarise to produce a 2I2O configuration or a 3I1O/1I3O monopole crystal, depending on the precise direction of the field misalignment. This is in contrast to the situation for applied [100] and [111] fields, for which the respective 2I2O order and 3I1O/1I3O monopole crystals are stabilised over a wide range of field angles around the [100] and [111] directions.

Figure 8a shows a Monte Carlo simulation of the magnetisation for an applied field tilted by 5.19° away from

[110] towards [100], which reproduces the experimentally observed behaviour well. There is an initial rapid rise in the magnetisation as the two spins which are not perpendicular to [110] are polarised by the dominant field component. At higher fields, the two spins unconstrained by the [110] component are polarised by the smaller [100] component to yield a 2I2O configuration, leading to a subsequent slower increase of the magnetisation and a saturation magnetisation which exceeds that expected for a perfectly aligned [110] field. Figure 8b shows a Monte Carlo simulation of the monopole density for an applied field tilted by 10.02° away from [110] towards [111]. The [111] component of the applied field couples to the two spins that lie perpendicular to [110] and the system orders into a 3I1O/1I3O monopole crystal, which generates an energetic pressure on the Ir domain walls. This plastically deforms the Ir domain ratio, opening a hysteresis similar to that observed for the [111] orientation, but closing at higher fields, because a smaller component of the applied field is directed along [111]. This behaviour of the monopole density reproduces the observed hysteresis in measurements of the resistance well.

VII. MECHANISMS LINKING MONOPOLE DENSITY AND RESISTANCE

The elementary excitations of the 2I2O ground state manifold of spin ices are effective sources or sinks of the (coarse-grained) magnetic field. According to the dumbbell model [6], the magnetic field of a pyrochlore spin ice is a combination of the Coulombic field of these monopoles and comparatively short-ranged quadrupolar corrections. Therefore, the magnetic scattering of electron spins off this field pattern is approximated well by charge–dipole scattering off the monopoles only. Furthermore, lattice distortions due to the frustrated magnetic structure generate effective electric dipoles on each pyrochlore tetrahedron hosting a monopole [7], which in turn results in *electric* charge–dipole scattering of the conduction electrons. It is reasonable to assume that these are the two dominant effects of the low-temperature holmium spin ice magnetism on the conduction electrons, and they are both charge–dipole type scattering off the emergent monopoles, in independent electric and magnetic channels. Note that we neglect the mobility of monopoles, which is reasonable given the very different dynamical time scales of Ho and Ir (milliseconds and femtoseconds, respectively).

Before we discuss these two scattering mechanisms in greater detail, we note that their effect is predicated on the scattering centres being randomly distributed in space. Near saturation in a strong [111] magnetic field, the pattern of magnetic moments (and hence of emergent monopoles and their electric dipoles) becomes increasingly ordered, and thus unable to scatter the conduction electrons. As a result, while the monopole density increases monotonically up to saturation in high magnetic fields, we expect the resistance to peak at an intermediate field value that optimises a dense but sufficiently random monopole density, before starting to decrease as the applied field is increased further up to saturation. This is indeed in agreement with our results in Fig. 3 in the main text. We note that this phenomenon is also discussed in a different but related context in recent work by Khomskii [8], who observes that the electric activity of the dipoles is strongly reduced in any monopole crystal, not only the fully magnetically ordered one in our case. This effect might be relevant for $Ho_2Ir_2O_7$ at lower temperatures, where moment fragmentation is more pronounced.

Let us now estimate the scattering rate of conduction electrons due to the two charge-dipole processes mentioned above. The transition matrix elements for a generic charge–dipole interaction are given by the Fourier transform of the dipole potential:

$$V(\mathbf{r}) = -C\,\frac{\hat{\mathbf{p}}\cdot\mathbf{r}}{r^3} \implies V(\mathbf{q}) = \int \mathrm{d}\mathbf{r} e^{-i\mathbf{q}\cdot\mathbf{r}} V(\mathbf{r}) = 4\pi i C\,\frac{\hat{\mathbf{p}}\cdot\mathbf{q}}{q^2}, \tag{1}$$

where $\hat{\mathbf{p}}$ is the unit vector parallel to the (electric or magnetic) dipole and C is a generic coupling constant, equal to $ep/(4\pi\varepsilon_0)$ and $\mu_0\mu_{\mathrm{B}}q/(4\pi)$ in the electric and magnetic cases, respectively, where q and p are the magnetic charge and electric dipole moments of the monopoles. Now, the scattering rate follows from general scattering theory [9] as

$$\tau^{-1} = \int \frac{\mathrm{d}\mathbf{k}'}{(2\pi)^3} W_{\mathbf{k},\mathbf{k}'}(1-\hat{\mathbf{k}}\cdot\hat{\mathbf{k}}'), \tag{2}$$

$$W_{\mathbf{k},\mathbf{k}'} = \frac{2\pi}{\hbar} n_{\mathrm{mp}}\,\delta\left(E(\mathbf{k}) - E(\mathbf{k}')\right)\left|V(\mathbf{k}'-\mathbf{k})\right|^2, \tag{3}$$

assuming the dispersion of conduction electrons is isotropic, and monopoles are dilute enough that electrons only scatter off one at a time. In Eq. (3), n_{mp} is the number density of scatterers (monopoles), and the delta function enforces elastic scattering. To make progress, we make two further working assumptions: (i) the dispersion of

conduction electrons is quadratic, $E(\mathbf{k}) = \hbar^2 k^2/(2m)$, with an effective mass m on the order of the bare electron mass; (ii) the orientation of dipoles, $\hat{\mathbf{p}}$, is uniformly distributed on the unit sphere. The latter is likely to hold for magnetic scattering, as the ordered iridium moments are small and their onset coincides with the metal–insulator transition, both of which suggest that the remaining conduction electrons are not spin-polarised. For electric scattering, the dipoles are oriented towards the minority spin of 3I1O and 1I3O tetrahedra [7]; numerical evidence suggests that these are essentially uncorrelated, apart from the [111] field-polarised limit. Given these assumptions, Eqs. (2, 3) can be evaluated to give

$$\tau^{-1}(k) = \frac{8\pi m^2 C^2}{3\hbar^4 k^2} n_{\mathrm{mp}} v, \tag{4}$$

where the group velocity is $v = \mathrm{d}E/(\hbar \mathrm{d}k) = \hbar k/m$. We note that the orientation of electric and magnetic dipoles in a given scattering process are likely uncorrelated (one belongs to the monopole, the other to the conduction electron), so the scattering rates due to these processes can simply be added (equivalently, the coupling constants C are to be added in quadrature). The correction to the resistivity can now be estimated from the Drude model:

$$\Delta\rho = \frac{m}{n_{\mathrm{e}} e^2}\langle \tau^{-1} \rangle = \frac{4\pi m^2 C^2 n_{\mathrm{mp}}}{\hbar^3 k_{\mathrm{F}} n_{\mathrm{e}} e^2}, \tag{5}$$

where n_{e} is the number density of conduction electrons and $k_{\mathrm{F}} = (3\pi^2 n_{\mathrm{e}})^{1/3}$ is the Fermi wave vector assuming a quadratic dispersion.

We can use Eq. (5) to estimate the carrier density in $Ho_2Ir_2O_7$. The magnetic charge of an emergent monopole is $q = 2\mu_{\mathrm{Ho}}/a_{\mathrm{d}} \approx 4 \times 10^{-13}$ Am [6], while the moment of the corresponding dipole has been estimated to be on the order of $p \sim 10^{-4} e$Å in $Dy_2Ti_2O_7$ [10]. It follows that the coupling constant C is on the order of 1 meVÅ^2 for both electric and magnetic scattering. In order to calculate the size of the resistivity hysteresis we need to determine the ratio A/l, where A is the cross-section of the sample perpendicular to the applied current and l is the distance between the voltage contacts. The sample has the shape of a truncated octahedron with 200 µm edge length and consequently the exact shape and dimensions of the cross-section between the contacts are very difficult to determine. At $T = 2$ K the experimentally measured resistance hysteresis is about $\delta R \approx 5$ mΩ at its widest point. Given that we estimate A/l to be $(6-9) \times 10^{-4}$ m, this corresponds to a resistivity hysteresis of $\delta\rho \approx (3-4.5) \times 10^{-6}\ \Omega\,$m. The corresponding width of the monopole density hysteresis in our simulations is $\delta n_{\mathrm{mp}} \approx 5$ nm^{-3}. These figures are consistent with a carrier density $n_{\mathrm{e}} \sim 10^{17}/\mathrm{cm}^3$ in (5): this is some six orders of magnitude below the carrier density of elemental metals, a sensible figure for a badly conducting semimetal like $Ho_2Ir_2O_7$.

It is important to point out that the metallic Fermi surface used in this estimate is an oversimplification of the electronic structure of $Ho_2Ir_2O_7$. The experimentally observed very slow increase of resistivity below the metal–insulator transition suggests that the system is not a band insulator (especially compared to $Nd_2Ir_2O_7$ [3, 11]), but rather a (semi)metal or a heavily doped semiconductor [12], or perhaps not even a Fermi liquid [13]. Nevertheless, we believe that our order of magnitude estimate remains reasonable even with these caveats in mind.

It is also interesting to contrast our result with previous calculations of magnetic scattering in pyrochlore iridates $Pr_2Ir_2O_7$ and $Nd_2Ir_2O_7$ [14, 15], which argue that their resistivity decreases as temperature (and thus monopole number) is increased. There are, however, several crucial differences between our present approach and these works. First, we consider changing monopole density (and spin correlations in general) at a fixed temperature, while in Refs. [14, 15] changes to spin correlators are intertwined with other effects of changing temperature in the self-energy calculation. Second, we focus on the coupling between the dipolar magnetic fields of the Ho moments and the Ir conduction electrons, rather than a short-range Kondo coupling studied in the prior work. Indeed, in $Pr_2Ir_2O_7$ and high-pressure $Nd_2Ir_2O_7$, rare-earth moments are substantially smaller than in $Ho_2Ir_2O_7$ and dipolar interactions between them are negligible, whereas these interactions dominate in the Ho case. Finally, in the same materials, no metal–insulator transition is driven by Ir antiferromagnetism [11]; the temperature dependence of the resistivity in $Ho_2Ir_2O_7$ is, on the other hand, dominated by such a transition.

VIII. SAMPLE GROWTH CONSIDERATIONS

Potential issues with sample stoichiometry were mitigated at the synthesis stage as follows. *Powder sample preparation*: we prepared the phase-pure polycrystalline $Ho_2Ir_2O_7$ powder sample with an excess of 5% IrO_2 powder in

order to compensate the evaporation loss due to high-temperature synthesis and used the final single-phase powder as a starting material for the crystal growth. *Single-crystal preparation*: during the crystal growth process, which was carried out at relatively low temperature, we have tightly sealed a second outer crucible with alumina wool to catch any IrO_2 evaporation, but we did not observe any sign of it. The cell parameter values of both powder (10.1792 Å) and single crystals (10.1801 Å) are consistent and so we do not expect any variation in the chemical composition. To confirm this, we have annealed the as-grown crystal in oxygen atmosphere and found no change in the cell parameter value. Moreover, both Ho and Ir have stable valence states, 3+ and 4+ respectively, so we believe that any significant oxygen variation in the crystal is unlikely.

A qualitative estimate of the sample quality can be deduced from various physical properties. The measured lattice parameters of our crystals of $Ho_2Ir_2O_7$ are in excellent agreement with the structural trend across the $RE_2Ir_2O_7$ family, see Refs. [16, 17]. Furthermore, it has been previously argued [18] that the effect of oxygen vacancies in non-Kramers spin-ice materials is to suppress the moments on the rare-earth sites. If this was happening in our crystals of $Ho_2Ir_2O_7$, it would be detectable via experimental measurements of the saturation magnetization along different crystallographic directions. On the contrary, the results we obtain are in very good agreement with the values expected from calculations made with the full Ho^{3+} moment.

SUPPLEMENTARY REFERENCES

[1] T.-h. Arima, Time-reversal symmetry breaking and consequent physical responses induced by all-in-all-out type magnetic order on the pyrochlore lattice, J. Phys. Soc. Jpn. **82**, 013705 (2013).

[2] Y. Yamaji and M. Imada, Metallic interface emerging at magnetic domain wall of antiferromagnetic insulator: Fate of extinct Weyl electrons, Phys. Rev. X **4**, 021035 (2014).

[3] Z. Tian, Y. Kohama, T. Tomita, H. Ishizuka, T. H. Hsieh, J. J. Ishikawa, K. Kindo, L. Balents, and S. Nakatsuji, Field-induced quantum metal–insulator transition in the pyrochlore iridate $Nd_2Ir_2O_7$, Nat. Phys. **12**, 134 (2016).

[4] L. Opherden, J. Hornung, T. Herrmannsdörfer, J. Xu, A. T. M. N. Islam, B. Lake, and J. Wosnitza, Evolution of antiferromagnetic domains in the all-in-all-out ordered pyrochlore $Nd_2Zr_2O_7$, Phys. Rev. B **95**, 184418 (2017).

[5] L. Opherden, T. Bilitewski, J. Hornung, T. Herrmannsdörfer, A. Samartzis, A. T. M. N. Islam, V. K. Anand, B. Lake, R. Moessner, and J. Wosnitza, Inverted hysteresis and negative remanence in a homogeneous antiferromagnet, Phys. Rev. B **98**, 180403 (2018).

[6] C. Castelnovo, R. Moessner, and S. L. Sondhi, Magnetic monopoles in spin ice, Nature **451**, 42 (2008).

[7] D. I. Khomskii, Electric dipoles on magnetic monopoles in spin ice, Nat. Commun. **3**, 904 (2012).

[8] D. I. Khomskii, Electric activity at magnetic moment fragmentation in spin ice, Nature Communications **12**, 3047 (2021).

[9] N. W. Ashcroft and N. D. Mermin, *Solid State Physics* (Holt, Rinehart & Winston, New York, 1976).

[10] L. Lin, Y. L. Xie, J.-J. Wen, S. Dong, Z. B. Yan, and J.-M. Liu, Experimental observation of magnetoelectricity in spin ice $Dy_2Ti_2O_7$, New J. Phys. **17**, 123018 (2015).

[11] K. Matsuhira, M. Wakeshima, Y. Hinatsu, and S. Takagi, Metal–insulator transitions in pyrochlore oxides $Ln_2Ir_2O_7$, J. Phys. Soc. Jpn. **80**, 094701 (2011).

[12] J. J. Ishikawa, E. C. T. O'Farrell, and S. Nakatsuji, Continuous transition between antiferromagnetic insulator and paramagnetic metal in the pyrochlore iridate $Eu_2Ir_2O_7$, Phys. Rev. B **85**, 245109 (2012).

[13] K. Wang, B. Xu, C. W. Rischau, N. Bachar, B. Michon, J. Teyssier, Y. Qiu, T. Ohtsuki, B. Cheng, N. P. Armitage, S. Nakatsuji, and D. v. d. Marel, Unconventional free charge in the correlated semimetal $Nd_2Ir_2O_7$, Nat. Phys. (2020).

[14] G.-W. Chern, S. Maiti, R. M. Fernandes, and P. Wölfle, Electronic Transport in the Coulomb Phase of the Pyrochlore Spin Ice, Phys. Rev. Lett. **110**, 146602 (2013).

[15] M. Udagawa, H. Ishizuka, and Y. Motome, Non-Kondo Mechanism for Resistivity Minimum in Spin Ice Conduction Systems, Phys. Rev. Lett. **108**, 066406 (2012).

[16] J. P. Clancy, H. Gretarsson, E. K. H. Lee, D. Tian, J. Kim, M. H. Upton, D. Casa, T. Gog, Z. Islam, B.-G. Jeon, K. H. Kim, S. Desgreniers, Y. B. Kim, S. J. Julian, and Y.-J. Kim, X-ray scattering study of pyrochlore iridates: Crystal structure, electronic, and magnetic excitations, Phys. Rev. B **94**, 024408 (2016).

[17] M. Subramanian, G. Aravamudan, and G. Subba Rao, Oxide pyrochlores — a review, Progress in Solid State Chemistry **15**, 55 (1983).

[18] G. Sala, M. J. Gutmann, D. Prabhakaran, D. Pomaranski, C. Mitchelitis, J. B. Kycia, D. G. Porter, C. Castelnovo, and J. P. Goff, Vacancy defects and monopole dynamics in oxygen-deficient pyrochlores, Nature Materials **13**, 488 (2014).
